# Automated classification of polyps using deep learning architectures and few-shot learning

**DOI:** 10.1186/s12880-023-01007-4

**Published:** 2023-04-20

**Authors:** Adrian Krenzer, Stefan Heil, Daniel Fitting, Safa Matti, Wolfram G. Zoller, Alexander Hann, Frank Puppe

**Affiliations:** 1 Department of Artificial Intelligence and Knowledge Systems, Julius-Maximilians University of Würzburg, Sanderring 2, 97070 Würzburg, Germany; 2 Interventional and Experimental Endoscopy (InExEn), Department of Internal Medicine II, University Hospital Würzburg, Oberdürrbacher Straße 6, 97080 Würzburg, Germany; 3 Department of Internal Medicine and Gastroenterology, Katharinenhospital, Kriegsbergstrasse 60, 70174 Stuttgart, Germany

**Keywords:** Machine learning, Deep learning, Endoscopy, Gastroenterology, Automation, Image classification, Transformer, Deep metric learning, Few-shot learning

## Abstract

**Background:**

Colorectal cancer is a leading cause of cancer-related deaths worldwide. The best method to prevent CRC is a colonoscopy. However, not all colon polyps have the risk of becoming cancerous. Therefore, polyps are classified using different classification systems. After the classification, further treatment and procedures are based on the classification of the polyp. Nevertheless, classification is not easy. Therefore, we suggest two novel automated classifications system assisting gastroenterologists in classifying polyps based on the NICE and Paris classification.

**Methods:**

We build two classification systems. One is classifying polyps based on their shape (Paris). The other classifies polyps based on their texture and surface patterns (NICE). A two-step process for the Paris classification is introduced: First, detecting and cropping the polyp on the image, and secondly, classifying the polyp based on the cropped area with a transformer network. For the NICE classification, we design a few-shot learning algorithm based on the Deep Metric Learning approach. The algorithm creates an embedding space for polyps, which allows classification from a few examples to account for the data scarcity of NICE annotated images in our database.

**Results:**

For the Paris classification, we achieve an accuracy of 89.35 %, surpassing all papers in the literature and establishing a new state-of-the-art and baseline accuracy for other publications on a public data set. For the NICE classification, we achieve a competitive accuracy of 81.13 % and demonstrate thereby the viability of the few-shot learning paradigm in polyp classification in data-scarce environments. Additionally, we show different ablations of the algorithms. Finally, we further elaborate on the explainability of the system by showing heat maps of the neural network explaining neural activations.

**Conclusion:**

Overall we introduce two polyp classification systems to assist gastroenterologists. We achieve state-of-the-art performance in the Paris classification and demonstrate the viability of the few-shot learning paradigm in the NICE classification, addressing the prevalent data scarcity issues faced in medical machine learning.

## Background

Colorectal cancer (CRC) is the second leading cause of cancer-related deaths worldwide [1]. This cancer develops from lesions inside the colon called polyps. However, not all colon polyps have the risk of becoming cancerous. Therefore, polyps are classified using different classification systems. After the classification, further treatment and procedures are based on the classification of the polyp. Since young physicians often do not have the necessary experience to make the correct decision reliably, computer-assisted procedures are being developed that can assist with the classification [2].

In the field of automated gastroenterological assistance systems, a significant area of research involves the detection of polyps using deep learning. Polyps are mucosal growths in various body parts, such as the intestine or stomach [3]. In some cases, unusual skin changes can become dangerous and even cancerous. Deep Learning object recognition methods such as CNNs detect and classify polyps automatically during examinations to assist endoscopists [4–6]. This may be beneficial for the future, to detect polyps more accurately by automated methods and to simplify or confirm the prognosis for the proper polyp treatment.

The polyp classification is essential as it helps the endoscopist decide on further treatment methods. For classification, different approaches are used to categorize polyps, such as schemes based on the shape (PARIS) [7] or based on the surface structure (NICE) [8]. The classification of polyps can give first insights into their dangerousness and the appropriate treatment options [7]. Furthermore, van Doorn et al. demonstrated a moderate interobserver agreement among Western international experts for the Paris classification system. Automated classification systems could help increase experts’ interobserver agreement on the Paris classification [9].

We consider the Paris and the NICE classification for our automated classification algorithms as they are the most commonly used classification in Europe. Furthermore, the Paris classification is recommended for documentation in the ESGE European Society of Gastrointestinal Endoscopy guidelines and it is also recommended to use advanced endoscopic imaging like NBI [10].

**Fig. 1:**
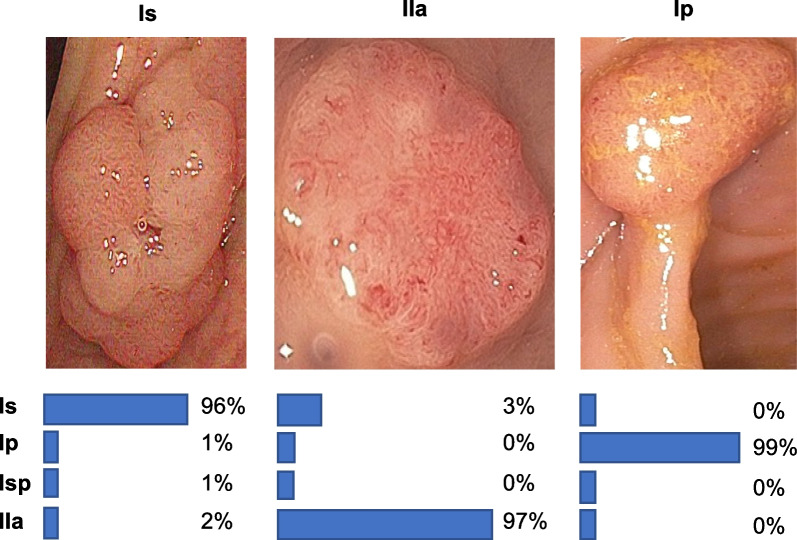
Automated Paris classification examples. This figure illustrated some classification examples of the polyp classification system on our data (EndoData) [11]. The percentage values show the confidence of the classification system

This paper shows therefore two automated classification networks. The first is classifying the polyp based on white light using the Paris classification scheme [7]. A two-step process is introduced: first, detecting and cropping the polyp on the image, and secondly classifying the polyp based on the cropped area with a transformer network. Figure 1 shows some example results of the Paris polyp classification system.

The second is the NICE classification, which is based on Narrow band imaging (NBI). NBI is a variation of endoscopy that uses blue and green light to enhance the visibility of surface patterns and texture of the mucosa. The presented NICE classification system is designed as a Deep Metric Learning based approach of few-shot learning to account for the data scarcity of NICE annotated images in our database.

In the following, the main contributions of the paper are shown:

1. We introduce a Paris classification system with state-of-art performance on clinical data.
2. We created a data set of polyp classification data to train and further enhance the models.
3. We present and validate a new approach for the automated NICE classification in data scarce scenarios leveraging few-shot learning.

Additionally, both polyp classification systems were publicly funded and developed by computer scientists and endoscopists in the same workgroup to ensure the high quality of the polyp classifications. In the next subsection a summary of the medical classification methods of polyps will be given. Furthermore, to overview existing work and properly allocate our paper to the literature, we describe a brief history from general polyp detection to state-of-the-art polyp classification with deep learning techniques.

### Medical backgroud

Polyps are small, fungal, or flat mucosal growths in various body regions, such as the intestines, stomach, uterus, or nose. The different-looking skin lesions most commonly occur in the stomach or intestines and affect in particular older people. They often appear after inflammation, leading to higher cell division in the mucosa. Additionally, polyps can become malignant or even cancerous due to unusual cell growth. Polyps can be divided into three types: hyperplastic, neoplastic, and inflammatory. While the hyperplastic and inflammatory types have no or lower risk of degeneration, the neoplastic polyps represent the most dangerous type of polyp [12]. These can increase the risk of cancer, especially as they grow. In order to prevent a severe progression due to polyps, repeated examination by an endoscopist through endoscopy is necessary. In this process, hollow organs such as the intestine are examined with an endoscope, a flexible tube equipped with a camera, and light.

**Fig. 2:**
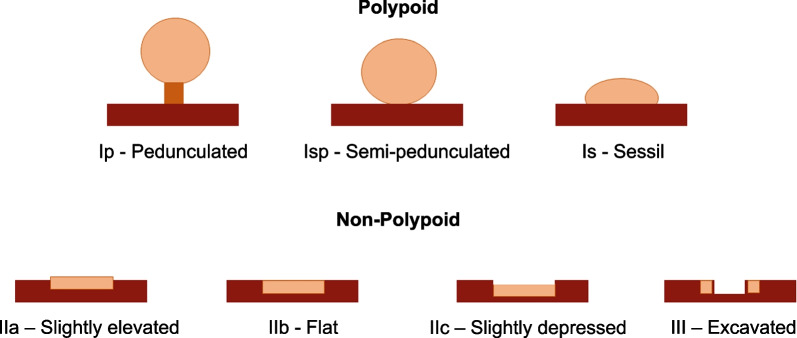
Subdivision of polyps according to Paris classification. Adopted from [7]

*Paris classification* In order to categorize polyps and to select appropriate treatment strategies, polyps are classified considering various aspects. One of the most widely used classifications is the Paris classification. Based on a Japanese classification scheme, the Paris classification characterizes the potentially high-risk polyps according to their shape [7]. Figure 2 visualizes the shapes of different polyps:

Type I polyps are referred to as elevated or polypoid. A distinction is made between the following polyp types:

- Ip Pedunculated
- Isp Semipedunculated
- Is Sessile

Type II polyps are described as flat. In addition, the following distinctions are made:

- IIa Slightly elevated
- IIb Completely flat
- IIc Depressed

Furthermore, lastly, type III describes the excavated form. Unlike type I, type II and III are not considered polypoid. A prognosis can be obtained through the Paris classification to conclude the type of polyp, and future treatment [7]. The Paris classification is sometimes given in the literature with a preceding 0 before the type. As the preceding is irrelevant to our approach, the leading zero is omitted for clarity.

Note, that the difference between the Paris classes Is and IIa is defined by the degree of elevation, with a protrusion in excess of 2.5 mm being defined as a polyp of Paris class Is [13]. To discriminate between the Paris classes Is and IIa, endoscopist experts normally rely on their instruments (such as the forceps) to provide a size reference [13]. Our data set and the open source data does not support ulcers (Paris type 0-III). Thus, a restriction towards non-ulcers lesions had to be made.

*NICE classification* The NICE classification is an established diagnosis scheme classifying polyps into three categories, which specify the most likely pathology ranging from benign hyperplastic to cancerous polyps deeply invading the mucosa underneath the polyp.

The scheme hereby utilizes the Narrow-Band-Imaging technology (NBI) to render the surface texture visible and to characterize the different polyp classes according to features such as the vessel patterns discernible on the polyp surface [14]. An overview of the different NICE classes,^1^ their characteristics and most likely pathology can be seen in Table 1.

The NICE classification has been well established as an informative feature for the classification of polyps [15, 16] and the clinical performance of the scheme, as well as the classification performance of human experts using the scheme, have been subject to numerous studies [14, 16]. In the treatment assessment guideline of the European Society of Gastrointestinal Endoscopy, the degree of submucosal invasion is a decisive criterion for the requirement of surgical removal of neoplastic polyps [17].

**Table 1:** Overview of the NICE categories

	Typ 1	Typ 2	Typ 3
Color	Same or lighter than background	Browner than background	Brown to dark
Vessels	None or isolated lacy vessels	Brown vessels around white structures	disrupted or missing vessels
Surface	Dark or white spots or homogeneous	Oval, tubular or branched white strucutres	amorphous or absent patterns
Likely pathology	hyperplastic	Adenoma	Deep submucosal invasive cancer
Examples	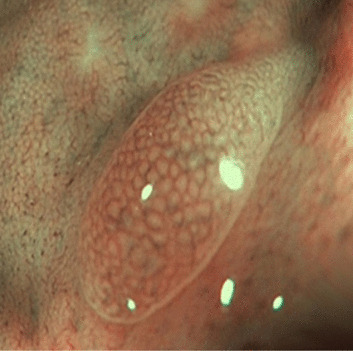	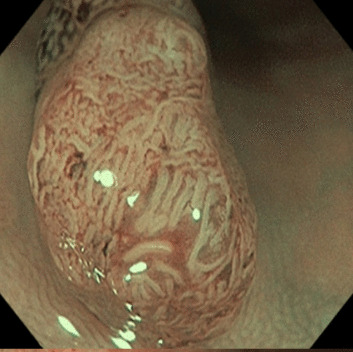	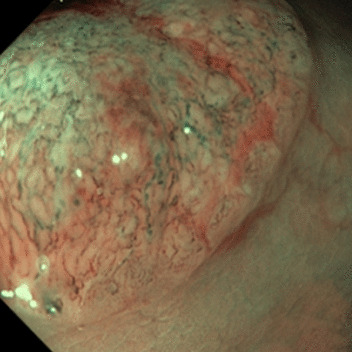

Adopted from Endoscopy-Campus GmbH$^{[1]}$

### A brief history of automated polyp classification

This section gives a brief overview of the current state of the art in automated polyp detection and classification research with respect to deep learning methods. Here, there are mainly two ways deep learning methods can be used to assist gasteroenterlogists with the assessment of polyps: For one, through the early detection of polyps in images or videos. For another, through the classification of polyps, in order to conduct a proper treatment analysis. Classifying polyps is based on various superficial features such as shape or structure. In this context, the detection and classification of polyps can be challenging due to numerous aspects.

Since this decade, deep learning has been the leading technology in developing computer-aided polyp detection. Most methods do use Convolutional Neural Networks (CNNs) for the detection of polyps. E.g Zhu et al. show a seven-CNN paired with a support vector machine (SVM) to detect anomalies in endoscopy images Zhu.2015. Another paper is the paper by Zhang et al., which presents a CNN for polyp detection and localization. They use a single-shot multibox detector that reused shifted information through max-pooling layers to achieve higher accuracy. They achieved a real-time detection speed of 50 frames per second (FPS) and an average accuracy of 90.4 [18]. Another idea from Bagheri et al. used sophisticated preprocessing involving the colors of the images to correlate the information to locate and segment polyps. In this way, their polyp detection achieved 97.7 % accuracy on the CVC-ColonDB dataset [19]. Another approach from Qadir et al. utilizes a two-step method. In the first step, they used a CNN that generated multiple regions of interest (RoIs) that are then used for classification. These proposed RoIs were compared with subsequent frames and their RoIs. The rationale of this method is that the frame in a video should be similar to the next frame, and this is to reduce the percentage of false predictions.

Sornapudi et al. also utilized region-based CNNs to localize polyps in colonoscopy images but in wireless capsule endoscopy (WCE) images. Therefore, the detection is not done in real-time. During localization, images were segmented and detected based on polyp-like pixels Sornapudi.2019. Currently, also transformer architectures are relevant for polyp detection. For example, a particular sparse autoencoder method called stacked sparse autoencoder with image manifold constraint has been used by Yuan and Meng [20] to detect polyps in WCE images. A sparse autoencoder is an artificial neural network commonly used for unsupervised learning methods [21]. Their approach achieved an accuracy of 98 % in polyp detection [20]. Another approach used transformers in combination with CNNs. Zhang et al. used the ability to view global information of the whole image through the attention layers of transformers and the detailed local detection of CNNs to segment polyps efficiently. They used a new fusion technique called BiFusion to connect the features obtained from the transformers and the CNNs. The method ran in real-time with 98.7 FPS [22].

Not only the localization of polyps represents a goal of computer-specific polyp research, but also the classification according to specific characteristics. For example, Ribeiro et al. used the feature extraction capability of CNNs to classify polyps into “healthy” (average) and “abnormal” (adenoma) classes using Kudo’s pit-pattern classification. Pit-pattern classification is a variant of categorizing types of polyps based on their surface structure [23]. The authors achieved an accuracy of 90.96 % by their classification using the CNN [24].

Using pit-pattern classification, a deep learning model was presented in the paper [25] to classify polyps into,,Benign,”,,Malignant,” and,,Nonmalignant. Here, the model was trained with a private data set and achieved a reliability of 84 %. Another popular polyp classification method using a CNN is used in [26]. Here, the authors used the Narrow-Band Imaging International Colorectal Endoscopic Classification (NICE for short) [8], similar to pit-pattern classification using surface features. Here, however, the polyps were additionally categorized by color or vascular structure and classified as polyp type 1 or 2. Thus, a preliminary prognosis can be determined whether the polyp is a hyperplastic or an adenoma tumor. For classification, the authors used a CNN with an SVM. The CNN was pre-trained on a non-medical data set to compensate for the lack of polyp data. They achieved an accuracy of nearly 86 % [26] with their proposed model.

Bryne et al. also used the NICE classification to characterize polyps. They classified them as hyperplastic or adenoma polyps. The authors created a CNN model for real-time application, which was trained and validated using only narrow-band imaging (NBI) video frames. In doing so, they achieved an accurate prediction of 94 % [27] on a sample of 125 testing polyps. Furthermore, Komeda et al. presented a specific CNN model to classify polyps into “adenoma” and “non-adenoma” polyps based on NBI and white-lighted images [28]. In the paper by Lui et al., another automatic classification model is presented to characterize polyps into endoscopically curable lesions and noncurable lesions based on the NBI and white-lighted images. The division into curable and noncurable is based on the types of polyps, such as hyperplastic or tubular. Lui et al. achieved an overall accuracy of 85.5 % with their model, with higher performance on NBI images [6]. In addition, Ozawa et al. used a CNN based on a single-shot multibox detector to detect and classify polyps. They trained and validated the model with a non-public data set and achieved a true-positive rate of 92 % during detection and characterized the detected polyps with an accuracy of 83 % [5]. In 2021, Hsu et al. considered the classification of polyp pathology using gray scale images and a customly designed classification network embedded into a detection and classification pipeline. They achieved an accuracy in the decision between neoplastic or hyperplastic polyps of $82.8 \%$ using NBI and $72.2 \%$ using white light [29]. A recent work in [30] considered the discrimination between hyperplastic and adenomatous polyps with different deep computer vision models, as well as with features provided by classic feature extraction algorithms, such as the Gabor filter. Their best model AlexNet achieved an accuracy of 96.4 %. An overview over the methods discussed here is presented in Table 2.

Regarding the NICE classification, our work can be considered as a polyp classification system categorizing the polyps into the classes hyperplastic and adenoma according the pathological interpretation of the NICE classes I and II. The same methodology has already been applied in the mentioned works in [26, 27], and we consider therefore the literature outlined in this section as the peer group of our work. But in contrast to most of the previous works, which learn a blackbox pathology classification system, we aim to factorize the pathological assessment by embedding the classifications into the previously introduced well-established classification schemes Paris and NICE , in order to make the pathology assessments more explainable. Instead of the direct prediction of the pathology, we make the prediction of the NICE and Paris class of a polyp to the subject of our study.

To the best of our knowledge, just one similar approach concerning the Paris classification has been published [4]. Bour et al. trained several well-known CNN architectures to classify polyps based on shape. The polyp images were divided into “Not Dangerous”, “Dangerous” and “Cancer” concerning the Paris classification. They labeled the Paris classes Is, Ip, Isp, IIa and IIb as “Not Dangerous”, class IIc as “Dangerous” and class III as “Cancer”. Their algorithms are trained on 785 images. They achieved an accuracy of 87.1 % with ResNet50 as backbone [4].

**Table 2:** Related methods occupied with the pathological assessment of colorectal polyps

Author	Year	Method	Data	Classification	Accuracy
Ribeiro et al. [24]	2016	custom CNN	Private	Healthy abnormal	90.96 %
Zhang et al. [26]	2016	CaffeNet	Private and [31]	hyperplastic adenoma	85.9 %
Bryne et al. [27]	2017	InceptionNet	Private	Hyperplastic adenoma	94 %
Komeda et al. [28]	2017	custom CNN	Private	Adenoma non-adenoma	75.1 %
Lui et al. [6]	2019	custom CNN	Private	Curable non-curable	85.5 %
Bour et al. [4]	2019	ResNet-50	Private	Not dangerous dangerous cancer	87.1 %
Tanwar et al. [25]	2020	VGG-16	Private	Benign Malignant Nonmalignant	84 %
Ozawa et al. [5]	2020	SSD	private	Hyperplastic adenoma	83 %
Hsu et al. [29]	2021	custom CNN	Private	Hyperplastic neoplastic	72.2 % (Weight light) 82.8 % (NBI light)
Chung-Ming et al. [30]	2022	AlexNet	Private	Hyperplastic adenoma	96.4 %

## Data and methods

The following chapter describes the methodology of this paper. The section starts with outlining the data sets used for the training process. Furthermore, the chapter involves one section for the methodology of the Paris classification and one section for the NICE classification. For the Paris classifcation, we use a two-step process involving first the detection of the polyp and the cropping of the image to the region of the detected polyp. In a second step, the cropped polyp is provided to a transformer architecture to classify it. For the NICE classification, we deploy a metric learning CNN pre-trained on a texture transfer learning and a self-supervision data set, which is subsequently fine-tuned on the extracted and cropped polyp images.

### Data sets

The current chapter will outline the data sets involved in the training of the NICE and Paris classification systems, which were compiled from different sources.

Due to the data sets containing only a subset of the required annotation types (NICE or Paris ), the sources for the two classification tasks only partially overlapped.

#### Paris 

For the training and evaluation of the Paris classification system, we used two data sets. The first is an open-source data set called SUN (Showa University and Nagoya University) colonoscopy video data set. The Sun Colonoscopy Video data set consists of approximately 160,000 images, of which approximately 50,000 images contain polyps. Other open source polyp data sets do mostly not attain the Paris classification type. The polyp images contain 100 different polyps annotated by experienced endoscopists from the Showa University. The distribution of the images among the polyp types can be found in the Table 3 [32]. Because only polyp images are needed for this work, polypless images were sorted out. Since the images in the data set are single video frames, images that were too small or blurred with unrecognizable content were removed manually to train the networks on recognizable images.

**Table 3:** Distribution of the images in the SUN$^[2]$ colonoscopy video data set [32]

Type of polyp	Number of polyps by type	Number of images by polyp type
Is	49 cases	23.154 images
Ip	8 cases	4.162 images
Isp	9 cases	4.684 images
IIa	34 cases	17.136 images

http://sundatabase.org/

The second data set is EndoData this was created by us at the University clinic of Würzbug [11]. In the next section the proccess of the data creation will be outlined briefly.

#### Own data creation

Previously we created a framework for faster endoscopic annotation. It involves a two-step process. First, a small expert annotation part and then a large non-expert annotation part [33]. Thereby shifting most of the workload away from the expert to the non-expert while retaining high data quality. We combined both tasks using AI to increase the annotation speed further. To speed up is up to 20 times compared to a traditional annotation tool. Thereby the process is divided between at least two people. First, an expert watches the video and labels some video frames to verify the object labeling. In the second step, a non-expert receives a visual confirmation of the given object and can label all following and preceding frames with AI support. In order to label individual frames, all of the frames have to be extracted from the video. Our system is then pre-selecting relevant frames automatically.

Thereby experts can focus on those keyframes. After the expert completes his annotations, the AI model gives the relevant frames. The AI is then detecting the polyps in the image and pre-labeling those. The non-expert can adjust and modify the AI predictions and use them for training the AI model.

In addition, the expert annotates the Paris and, if possible, the NICE classification [7], the size of the polyp and its position, as well as the start and end image of the polyp and a box for the non-expert annotators. Afterward, Endodata [11] is filtered and the relevant Paris and NICE classification parts are extracted to create the final data set used in this paper.

We assembled a team of experienced gastroenterologists and medical assistants to create this data set. The EndoData data set contains 79,625 images with Paris classification involving 364 polyp sequences. The polyp sequences were selected in high quality because we usually annotated only the first 1–3 s of polyp appearance, which is critical for polyp detection in a real clinical scenario. We only used the NBI light images and videos from the Olympus processor for the NICE classification.

#### NICE

As the SUN database does not contain NICE class annotations and little data with a direct NICE annotation is publicly available, only a very limited data set of NICE annotated colorectal polyps was available for this study, comprising the images of not more than 61 different polyps. The data set contained polyp images of two different sources, namely the examples provided for the different NICE classes curated on the Endoscopy Campus^2^ and images extracted from the closed source endoscopic data set of the University of Würzburg, which were annotated by an expert gastroenterologist. As the data from the Endoscopy Campus provides only a single image per polyp and the usable frames of a specific polyp in the closed source data were nearly identical, the data set has been constructed to contain only a single image for each polyp.

Due to a lack of data, the third category of the NICE classification scheme has been dropped and the study focuses on the prediction of the first two classes, corresponding in the canonical interpretation to the two classes of hyperplastic and adenomatous polyps. Similar restrictions have already been made in other studies, such as in [2], discussed in the related work of this study. The data set comprises overall 27 images of class NICE II polyps and 34 images of class NICE I.

Due to the data set containing only a single image per polyp, the splits of the data set were disjoint concerning the contained polyp specimens and did not introduce any immediate or latent correlations between training and testing data.

As preprocessing measures, the images were cropped to the polyp region and down- or upsampled to a common shape of $224 \times 224$. The images have not been made subject to further preprocessing methods.

### Paris classification

The first classification method will focus on the Paris classification using white light endoscopy. The following subsection will illustrate the automated NICE classification.

*Reason for leaving out classes of the Paris classification*

As explained earlier polyps are divided into polypoid and non-polypoid in the Paris classification. Type I polyps are polypoid, and type II and III polyps are non-polypoid. Due to the composition of available data, only Is, IIa, Ip, and Isp forms were considered and used to classify polyps. Here, Is denotes the sessile type, IIa the flat raised polyps, Ip a pedunculated form, and Isp the semi-pedunculated polyps [7]. We do not have any data examples for the Paris categories IIb, IIc, and III in our data and the open source SUN data set. This may be due to the acquisition of most of the data from screening coloscopies where Paris types IIb, IIc and III are very rare. Therefore we had to remove those categories in our classification model. By classifying polyps into different types, it is also possible to make statements about the probability of a polyp being cancerous. In one study, it was shown that certain types in the Paris classification can lead to an increase in submucosal invasion. This correlates with a greater risk of developing lymph node metastases from polyp disease in the stomach, which may lead to a poorer prognosis. This revealed that polypoid type I (57 %) and types IIc (37 %) and III (40 %) had a higher risk of submucosal invasion. In comparison, forms IIa and IIb (29% and 20%) showed a lower probability of [7, 34].

Since the images in the data set are single video frames, images that were too small or blurred with unrecognizable content were removed manually to train the networks on recognizable images. Finally, the obtained images were prepared for the models and examined with respect to resolution.

**Fig. 3:**
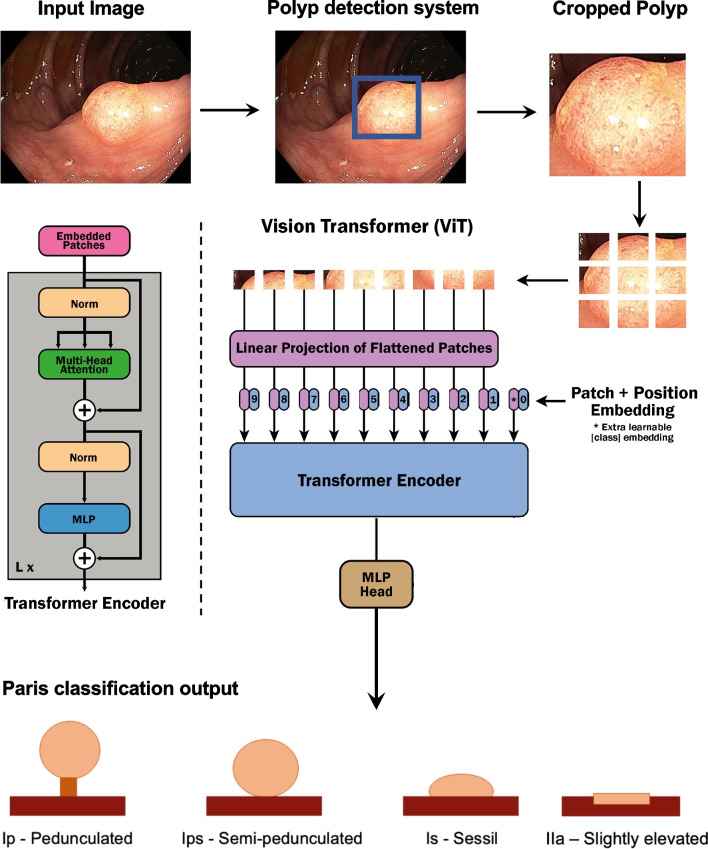
Structure of the polyp classification system. Adopted from [35]. Polyp images are from our data (EndoData) [11]

The problem of the Paris classification is to accurately categorize polyps in medical images into the different subtypes based on shape. This is important for effective diagnosis and treatment of colonic polyps, which can lead to colorectal cancer if not detected and managed early. Current methods for polyp classification face challenges in terms of accuracy and inter-observer variability, and there is a need for a more robust and reliable solution. The aim is to develop a system that can accurately classify polyps based on the Paris criteria, and provide consistent and reliable results for clinical use.

Figure 3 outlines the structure of our polyp classification system. At the left site, you can see the photo taken from the endoscope processor, which was done after finding the polyp. This image is the input image to our system, and the next step is the polyp detection system. For the polyp detection system, we used ENDOMIND-Adcanced [11], which is a polyp detection system. The system was developed by us using a post-processing technique based on video detection to work in real-time with a stream of images. This allows leveraging the incoming stream context of the endoscope while maintaining real-time performance. The system, therefore, can predict a bounding box surrounding the polyp. In the next step, the image is cropped at the box corners. The background, which is unnecessary for the classification, is cropped so that the polyp is better processed by the following classification step. In the classification step, the resulting polyp image is inserted into the Vision transformer (ViT) [35].

The use of transformers in computer vision is a relatively new field but is a significant competitor to CNNs. The paper Vision Transformer (ViT) introduces the use of transformers in the image processing domain without using a CNN. The Vision Transformer is based on a classical transformer for NLP, which has been adapted for the computer vision task. The input image is brought into fixed-size image sections, also called patches, as visualized in Fig. 3. Then, the image patches are passed to the transformer as a sequence, like a sentence sequence. The image sections are converted into computable vectors for the transformer using the patch embedding layer. Furthermore, the positions of the image sections are marked by Positional Embedding, as in a classical Transformer. In addition, a learnable classification token is added. The prepared sequence is then passed to one or more standard Transformer encoders. Unlike the classical transformer, the ViT model does not have a decoder, but a MLP head linked to the previous layers for classification [35, 36]. For pretraining the vision transformer, a large data set is used. For fine-tuning, the pre-trained classification part, the MLP Head, is then removed and replaced by a feed-forward layer specified for the desired task and adapted [35].

The developers of ViT provide three different transformer models for image classification: ViT-Base (12 encoder layers), ViT-Large (24 encoder layers), and ViT-Huge (32 encoder layers), which are available in the following variants: ViT-B/16, Vit-B/32, Vit-L/16, ViT-L/32 and Vit-H/14, the latter not being provided. The trailing number represents the number of image sections during processing. The models were pre-trained with the ImageNet-21k data set [35].

For our classification model, we used the ViT-L-16 model. In the end, the transformer outputs a number between 0 and 3, corresponding the Paris classification.

*Benchmark models*

We used two CNN benchmark models to contest our Paris classification system:

The first is Big Transfer (BiT). It uses the principle of transfer learning, in which a convolutional neural network is pre-trained on a huge data set. The pre-trained network is then selected and re-adapted to the relevant problem, also known as finetuning. The tranfer learning principle is used to compensate for deficiencies in training and testing examples in a data set for training a CNN. Transfer learning can be particularly relevant in the medical classification domain, as many medical data sets contain only a small number of data [37].

The second is Efficient Net. Convolutional Neural Networks have dominated the field of computer vision for years due to their good performance. However, CNNs are dependent on the resources available to build and scale the neural networks. Due to limited resources, scaling a neural network is one of the core problems that Google (Research) is trying to solve with its CNN models called EfficientNet [38]. Scaling a Convolutional Neural Network refers to adjusting certain dimensions that can lead to higher accuracy. Common model scaling is performed on the depth, the width of a CNN, or the resolution of an input image. Here, the depth of a model refers to the number of layers in a Convolutional Neural Network. Width is the number of channels in a layer, while resolution refers to image ratios such as height and width [38].

### NICE classification

The NICE classification is a widely used method for categorizing polyps based on their morphological features. However, there is a need for improvement in the reliability and consistency of polyp classification using the NICE system. The objective is to develop a robust and efficient NICE polyp classification system that can accurately and consistently categorize polyps based on the NICE criteria.

The data situation faced in the NICE classification outlined in the preceding sections is frequently encountered in artificial intelligence, but is a particularly ubiquitous problem in the medical domain of machine learning: Few data sets are made publicly available, but retained as private resources, the amount of data is limited, especially for rare conditions and cases, and the expertise requiring annotations are costly and time-consuming to acquire. This core issue of artificial intelligence has been subject to inquiry in recent years and the prolific branches of zero-shot and few-shot learning have emerged as potential remedies for the data scarcity issues in many machine learning domains [39]. The former refers to algorithms attempting classifications without having been trained on an example of the target classification task, while the latter refers to strategies in which the availability of a few training examples is leveraged for the fine-tuning of zero-shot classification systems.

few-shot learning (FSL) is an active and promising research branch aiming to cross the chasm between the learning behavior of current machine learning systems and that of humans, who achieve high generalization capabilities from a few examples.

Given the data situation faced in the NICE classification of this study, we will explore the performance of FSL approaches in the context of polyp classification. The following section will provide a brief outline of the relevant background of FSL.

#### Few-shot learning

The FSL literature comprises a large stock of different strategies and philosophies to approach the data scarcity issue. The approaches range from the intensive application of data augmentation methods expanding the data set in order to enforce desired invariances in the classification model, transfer learning strategies and even complex meta-learning algorithms, which are trained to provide parameterizations for a model given a few, or even only single example of the target task [40].

A popular and well-established approach in the transfer learning branch of FSL is embedding learning [41], in which an embedding model $f: R^m \rightarrow R^n$, where $n \ll m$, is trained, such that task-specific notions of similarity between inputs, manifest as trivially quantifiable similarities between their latent representations generated by the model *f*. In the desired structure of the latent space, the samples of classes do not form a complex manifold but form clusters, allowing distance metrics, such as the euclidean or the cosine distance, to quantify the similarity and class affiliations of samples. A latent space exhibiting such structural properties might then allow the construction of simple class discrimination hypotheses, which are within reach with little data available for the target task. Frequent choices for hypothesis are as simple as a k-nearest neighbour classification [39, 42].

The embedding model *f* can be learned through transfer learning from a task-unrelated but extensive data set and might subsequently be fine-tuned to the target task data depending on the specific amount of data available.

There are many strategies for training the embedding model *f*, such as the Matching Networks [42] or the Prototype Networks [43]. In this study, we selected concepts of Deep Metric Learning to enforce the desired structure on the latent embedding space.

#### Deep metric learning

The field of Deep Metric Learning is occupied with the training of encoder models, which enforce the previously discussed properties of the latent space in order to provide a semantic metric in conjunction with a specified distance measure [44].

In the field of metric learning, the approach of Siamese networks is an established training paradigm for the encoder. The concept of Siamese networks has first been considered in the field of signature verification [45], but has since then been ported to CNNs and numerous applications including few-shot scenarios [46].

Conceptually, a siamese network comprises a neural network and a weight-sharing clone, which are subsequently trained on pairs of data points, which might constitute a positive pair, demonstrating semantic similarity or a negative pair demonstrating semantic dissimilarity. The neural network and its clone are then trained to produce embeddings with small in the former, respectively high distance in the latter case w.r.t. a selected distance metric.

Hoffer et al., however, realized that the standard approach of the siamese neural network produces sub-optimal results, if the metric is subsequently to be used for classification tasks, as the minimization and maximization of distances between positive and negative pairs does not necessarily lead to the intra-class distances being smaller than inter-class distances [47]. Hoffer et al. proposed to extend the siamese network to a triplet neural network, which comprises three weight-sharing clones of a neural network and is trained on triplets of data points consisting of an anchor instance *x*, a positive $x^+$ and a negative instance $x^-$ exemplifying semantic similarity and dissimilarity to the anchor instance respectively [47].

The training of the network *f* is then designed to enforce a class-consistent distance metric $\Vert f(x), f(x^+)\Vert _{\mathcal {D}} < \Vert f(x), f(x^-)\Vert _{\mathcal {D}}$ for a metric $\mathcal {D}$ and for all triplets $(x, x^+, x^-)$.

**Fig. 4:**
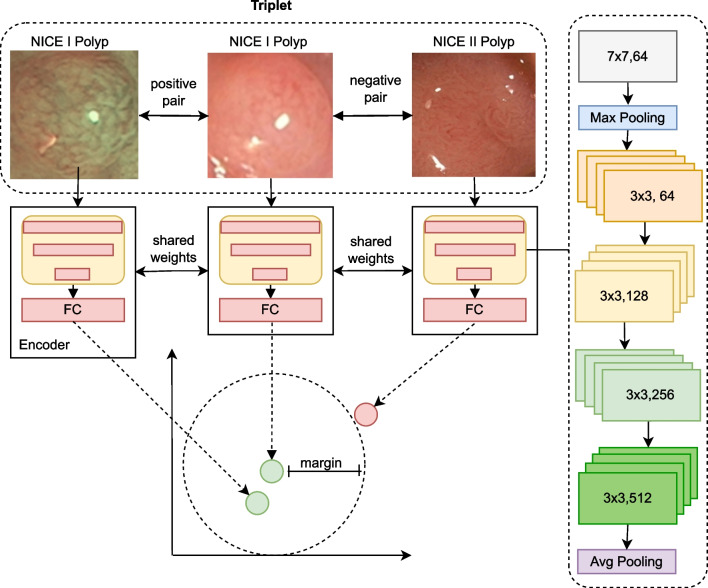
Illustration of the network architecture of a triplet network. According to equation 1, the distance between the vectors of the negative pair is increased to the selected margin

A variety of losses for the triplet network has been proposed for specific scenarios (such as in [48, 49]), but they are generally based on variations of the contrastive loss for siamese networks. For this study, an adaption of the contrastive triplet loss given in [50] is deployed:

1 $$\begin{aligned} \mathcal {L}_{triplet}(x, x^-, x^+) = \Vert f(x), f(x^+)\Vert _{\mathcal {D}} + max(0, m - \Vert f(x), f(x^-)\Vert _\mathcal {D}) \end{aligned}$$

where *m* is a margin parameter, which limits the total decrease in loss value achievable by high distances between the negative pair of the triplet and thus prevents network degeneration tendencies. The concept is illustrated in Fig. 4.

#### Considered approaches and methodology

With the background regarding few-shot and deep metric learning outlined, this section will discuss the methods in more detail and provide technical aspects regarding the selected hyperparameters used.

Specifically, we will deploy the triplet neural network concept with the loss given in equation 1, with a margin of $m = 20$ and with the metric being the l2-norm.

For the encoder itself, a member of the ResNet-family, ResNet-18, has been selected as the feature extraction backbone, as no performance gains were achievable using the larger conspecifics such as ResNet-50. The downstream classification layer of the ResNet-18 has been truncated and substituted with a single feedforward encoding layer embedding the average pooled feature map of the backbone into a 64-dimensional latent space.

The encoder has been pre-trained on a transfer learning data set and has been fine-tuned with the available polyp data. Importantly, the fine-tuning did not operate on the classification performance directly, but improved the consistency of the learned metric w.r.t. to the NICE data set using again the triplet loss of equation 1. For the fine-tuning the triplets were formed according to the NICE class affiliation. The fine-tuning scenario is depicted in Fig. 4.

During the fine-tuning, the training data set, comprising $75\%$ of the available labeled polyp image, has been expanded using a data augmentation process.

As augmentations, random flips along all image axes, as well as random modifications of image hue, contrast, brightness and saturation, have been implemented. The fine-tuning and model selection were subject to an early stopping strategy facilitated by $25\%$ of the train set held back for validation purposes. A single training epoch consisted here of 100 randomly generated triplets.

The embeddings have finally been tested in conjunction with different classification strategies, namely nearest-neighbour (referred to as 1-nn), the smallest average distance (referred to as centroid), or the Support Vector Machine (SVM) [51] equipped with the radial-basis-function kernel. For the 1-nn and centroid approach, the embedded images of the training set served as the latent space population for the test data classification. In the case of the SVM, the embeddings of the training data were used to fit the Support Vector Machine.

In this study, we are particularly interested in the effects of the pretraining and the considered transfer learning data set. We will therefore consider the usage of an out-of-domain, labeled data set and a within-domain, self-supervision-based data set for the pretraining.

*Supervised pretraining* The challenge of transfer learning is to select a transfer learning data set where the learned notions of semantic similarity are to a large degree aligned with the similarity notions of the target domain, especially if the potential transfer learning data sets exhibit significant domain gaps to the target data regime (such as endoscopic videos).

As the NICE classification scheme is largely based on surface patterns and the textures of polyps [8], we opted in this study for the texture classification data set Describable Texture Data set^3^ (DTD for short) [52].

The DTD data set provides a texture database containing 5640 images belonging to 47 different classes of human-distinguishable textures.

As the encoder model is trained with the loss given in equation 1, the construction of triplets is a mandatory preprocess. While the literature has discussed the usefulness of the mining of informative triplets both for the efficiency of training and quality of the discrimination capability (for instance [53]), for the study at hand, the triplets have been randomly mined with positive pairs originating from the same texture classes and negative image pairs from different. Since the DTD data set is a multilabel data set, with some training instances displaying characteristics of different textures simultaneously, the triplet mining selected the negative instances $x^-$ as completely class-disjoint with the anchor instance *x*.

As a measure to reduce the domain gap between the DTD data set and the polyp images and to provide the encoder with an organic invariance towards highlight corruptions, a preprocessing step has been implemented by grafting random specular highlights extracted from the SUN data set with the detection algorithm of Arnold et al. [54] onto the DTD images. The effect of this preprocessing step will later be discussed in an ablation experiment.

*Self-supervised pretraining* An alternative approach for pretraining neural networks is the strategy of self-supervised learning. The advantage of self-supervised learning algorithms is their defining independence of labeled ground truth data resulting from their eponymous capability to produce their supervision signal.

A further advantage of the self-supervised approaches is the possibility of tapping into available domain-related data sets. While these data sets lack the relevant ground truth annotation, they might still allow for a pretraining of networks exhibiting smaller domain gaps concerning the target tasks.

Especially in the medical domain, the independence of labeled training data of self-supervised approaches can therefore enable the leveraging of as much of the available medical data as possible, which is often idiosyncratic (endoscopic images, X-ray scans, etc.).

At a high level, the self-supervised approaches can be divided into generative and discriminative approaches [55], with the former category comprising strategies such as AutoEncoders [56] and the latter comprising again contrastive approaches [55].

**Fig. 5:**
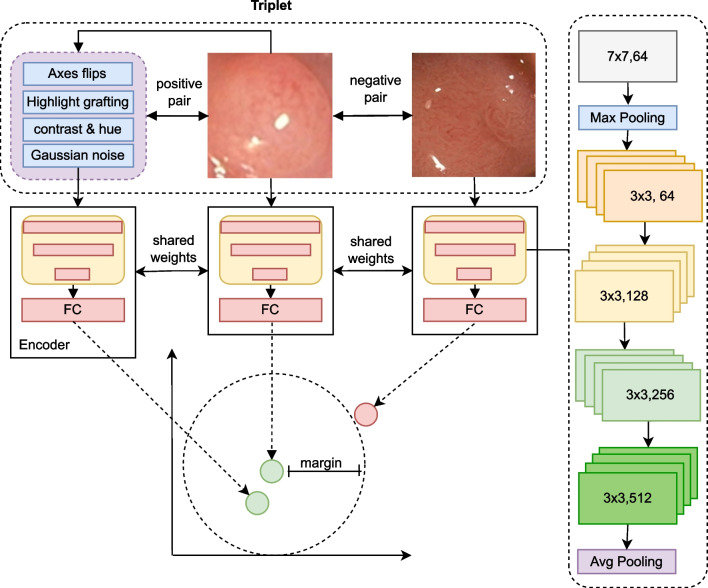
Illustration of the network architecture of a self-supervised triplet network. According to equation 1, the distance between the vectors of the negative pair is increased to the selected margin. The positive pair is built from an image and an augmented view of it

The fundamental insight and rationale of using contrastive approaches in self-supervision is that the representations of images and heavily augmented versions of them should be close in the latent space. In contrast, the distance to entirely unrelated images should be more significant. Hence, the self-supervision is again formulated as a triplet metric learning application and the network is enticed to embed the images into representations, which encode features, which are for one invariant towards all applied augmentation methods and for another discriminative towards other images. The concept of the self-supervised training of the encoder is illustrated in Fig. 5.

This latter discriminative approach has been used as a self-supervised pretraining strategy for the study. The already introduced SUN data set has been used as a source of endoscopic images. For the training, only images containing polyps have been used, which were cropped to the polyp regions and scaled to a common shape of $224 \times 224$. Only a fraction of the images in the SUN data set have been deployed for training. The roughly 50,000 polyp images have been condensed into a set of approximately 2500 images, which were extracted using an ORB-feature matching based temporal downsampling of the video sequences proposed in [57]. Utilizing the feature matching, the videos were decomposed into a sequence of scenes, out of which the sharpest frames were automatically selected.

As augmentation steps, random flips along all image axes, histogram altering modifications of image hue, contrast, saturation and brightness, and a random gaussian noise have been applied to the images. To further avoid encoding the prevalent specular highlights in the images as a kind of fingerprint, random specular highlights have been grafted onto the images, which have been again extracted from endoscopic images with the specular highlight detection algorithm of Arnold et al. [54].

## Results

In this section, we present the results of our two polyp classification systems. We will consider the two subsystems for the Paris and NICE classification separately, starting with the latter classification problem.

### Nice classification

The evaluation of the NICE classification system will consider the classification performances of both the full system, comprising the pre-trained encoder network and the subsequent fine-tuning, as well as the stand-alone pre-trained encoder without subsequent fine-tuning.

Beyond that, a range of classification algorithms applied to the embedded polyp images will be considered.

Finally, some design choices will be revisited through ablation experiments.

The experimental design, which has been outlined in the preceding section, will here be briefly recapitulated concisely: Roughly $75\%$ of the data has been used for the fitting of the classification algorithm and optionally for the fine-tuning of the encoder network. The test data comprised a class-balanced set of roughly $25\%$ of the polyp data. Due to the nature of the data set containing only one image per polyp specimen, the train and test set did not overlap concerning the contained polyp specimens.

As the data split is not negligible in the case of small data sets, we report the average performance of the system across 100 random train/test data splits and the $90\%$ confidence intervals. We expected the confidence intervals to be rather large, as the small data set was unlikely to support a completely split-robust decision boundary.

The same train/test splits were used for all experiments. Note at this point, that due to the nonlinearity of the also reported F1-score, the average F1-score is not necessarily equal to the F1-score of the average precision and average recall.

#### Classification without fine-tuning

This section considers the classification results without a fine-tuning step of the encoder model. The not fine-tuned models were considered to elucidate, how or if at all the differences in the pretraining strategy would manifest in the direct classification performance. The results are given in Table 4.

While the Support Vector Machine is the most complex discriminator considered, it displays better performance by a large margin compared to the nearest neighbour and average distance classifier, which indicates, that the two NICE classes are not completely separated in the latent space. Another point of view on this circumstance can be gained in Table 5, where the inter- and intra-class variances are reported for the embeddings of the differently pre-trained encoders. Table 5 shows, that the not fine-tuned DTD encoder fails at producing a compact cluster for the NICE II class. The encoder trained on the SUN images using self-supervision produces more consistent embeddings for the polyp images, which is also reflected in its better performance in the classification in Table 4. We attribute this difference in performance to the domain gap between the polyp images and the images in the DTD data set.

**Table 4:** Classification evaluation results not fine-tuned versions of the model pre-trained on the DTD data set and an endoscopic data set using self-supervison

Model	Classification	Acc	Pre	Rec	F1
DTD	1-nn	68.13 (± 14.21)	69.48 (± 12.25)	68.13 (± 14.21)	68.8 (± 15.2)
	centroid	67.69 (± 10.81)	72.55 (± 11.37)	67.69 (± 10.88)	70.0 (± 12.6)
	SVM	68.64 (± 11.83)	71.93 (± 10.13)	68.64 (± 11.83)	70.1 (± 13.7)
self-sv.	1-nn	65.34 (± 10.50)	65.91 (± 11.43)	65.34 (± 10.50)	65.7 (± 12.9)
	centroid	65.38 (± 15.32)	67.72 (± 14.71)	65.38 (± 15.32)	66.5 (± 15.9)
	SVM	72.55 (± 13.82)	73.95 (± 12.61)	72.55 (± 13.82)	73.3 (± 14.7)

The table shows the average scores for 100 random training/test splits with $90\%$ confidence intervals

**Table 5:** Intra- and interclass variances of the non fine-tuned polyp image embeddings of the models trained on the DTD data set and endoscopic data set with self-supervision

Model	Intra NICE I	Intra NICE II	Inter
DTD	0.62	1.27	1.0
self-supervision	0.88	0.85	1.0

The interclass variance is normalized to 1

#### Classification with fine-tuning

This section considers the performance of the two encoder systems with a fine-tuning step. To that end, the train data of the polyp images have been used to produce triplets with negative and positive triplet components selected according to their NICE class affiliation. Besides, a set of augmentations has been applied to the triplet images, encompassing random flipping along all image axes and heavy histogram modifying operations acting upon hue, contrast, brightness and saturation of the images. The training used early stopping facilitated by a held-out validation part of the train set. The results are reported in Table 6. Fine-tuning increased the top performance for both pretraining strategies, especially for the model trained on the DTD data set, which exhibits the overall top performance. We attribute this strong increase in performance of the DTD trained model to closing the domain gap between the DTD and polyp images. The results of the DTD trained encoder vis-á-vis the fine-tuned self-supervision system indicate however, that the pretraining on the texture data set bestowed the model with a superior and better generalizing feature extraction capability, which constituted a better initialization for the refinement of the representations.

The SVM classification performed well for both pretraining strategies in relative terms, with the smallest average distance producing even slightly better results on the DTD pre-trained model.

**Table 6:** Classification evaluation results in fine-tuned model versions pre-trained on the DTD data set and an endoscopic data set using self-supervision

Model	Classification	Acc	Pre	Rec	F1
DTD	1-nn	75.31 (± 9.41)	75.94 (± 8.63)	75.31 (± 9.41)	75.7 (± 9.8)
	centroid	81.39 (± 8.53)	82.05 (± 8.61)	81.39 (± 8.53)	81.7 (± 8.4)
	SVM	81.34 (± 8.74)	81.52 (± 8.39)	81.34 (± 8.74)	81.0 (± 8.6)
self-sv.	1-nn	71.59 (± 8.74)	75.09 (± 8.13)	71.59 (± 8.74)	73.3 (± 9.5)
	centroid	68.88 (± 8.45)	70.30 (± 8.82)	68.88 (± 8.45)	69.6 (± 9.7)
	SVM	75.04 (± 8.59)	75.24 (± 8.38)	75.04 (± 8.59)	75.1 (± 8.3)

The table shows the average scores for 100 random training/test splits with $90\%$ confidence interval

In summary of the results of the preceding two experiments and following the methodology of [27], who base their pathology assessment of polyps on the classes I and II of NICE , we conclude, that the here presented FSL model displays performances comparable to the results reported in the literature reviewed in the related work section of this study, despite the very limited amount of data available and the partially suboptimal acquisition of the images (without the NBI mode activated).

Moreover, we conclude that in the case of sufficient fine-tuning data being available, it is advantageous to conduct the pretraining on transfer learning data sets, in which the alignment of the presumed feature extraction capabilities learned from the data set, and the required capabilities for the target task is easier to foresee, as it has been the case with the texture DTD data set. While a smaller domain gap proved advantageous in our experiments (refer back to Table 4), when fine-tuning was not conducted, the self-supervision primed the encoder model in a way that allowed only for a minor refinement of the embeddings, which could be converted only into a small gain in performance, before the overfitting to the training data set in. Furthermore, the fine-tuning consolidated the confidence intervals significantly across the considered data splits.

#### Ablation considerations

This section will discuss the effect and influence of a few design choices made throughout the description of the NICE classification model. The average results of the 100 considered random train/test splits are reported.

First, we consider the influence of the data augmentation applied on the training data during fine-tuning. The results are presented in Table 7. While the augmentation yields for both pretraining strategies the best models concerning the F1-score, the performance difference is only small. The main incentive for introducing the training augmentation in the first place was to ensure that the classification was not based on spurious correlations in the small data set. But as the not augmented runs did not produce better results, even slightly worse, it is concluded that this worry was not justified, to begin with.

**Table 7:** Effect of augmentation during fine-tuning for differently pre-trained embedding models

Model	Augm.	Acc	Pre	Rec	F1
DTD	N	80.73 (± 8.48)	**82.05** (± 8.74)	80.73 (± 8.48)	80.5 (± 8.2)
	Y	**81.34** (± 8.74)	81.52 (± 8.39)	**81.34** (± 8.74)	**81.0** (± 8.6)
self-sv.	N	74.03 (± 8.97)	**76.13** (± 8.49)	74.03 (± 8.97)	75.0 (± 8.1)
	Y	**75.04** (± 8.59)	**75.24** (± 8.38)	**75.04** (± 8.59)	**75.1 **(± 8.3)

The classification was performed using a Support Vector Machine. The table shows the average scores for 100 random training/test splits with $90\%$ confidence interval. Bold values are indicating the highest value of a column

Finally, we consider the effect of the augmentation strategy of grafting random specular highlights on the images of the DTD data set during the pretraining of the encoder. In this experiment, we analyze whether accounting for the invariance towards these image corruptions can be fully substituted through fine-tuning and how it affects the not fine-tuned models. To that end, we considered an encoder trained on the DTD data set without the highlight augmentation grafting vis-á-vis the previous encoder in both the fine-tuning and no fine-tuning setting. The results are reported in Table 8. As the results indicate, the effects of the highlight-grafting operation depend heavily on the subsequent fine-tuning. While the augmentation increases the performance in all cases, the fine-tuning can catch up with the invariance towards the specular highlights. However, the non-finetuned model without the augmented pretraining suffers to a larger extent from interferences of the image corruptions.

**Table 8:** Effect on the specular highlight grafting augmentation during pretraining of the encoder with the DTD data set

Model	finetuning	highlight grafting	Acc	Pre	Rec	F1
DTD	Y	N	80.44	80.51	80.44	80.3
	Y	Y	**81.34**	**81.52**	**81.34**	**81.0**
	N	N	65.81	63.86	65.81	64.7
	N	Y	**68.61**	**71.92**	**68.61**	**70.1**

The average performance on 100 random train/test splits is reported. Bold values are indicating the highest value of a column

#### Error analysis

**Fig. 6:**
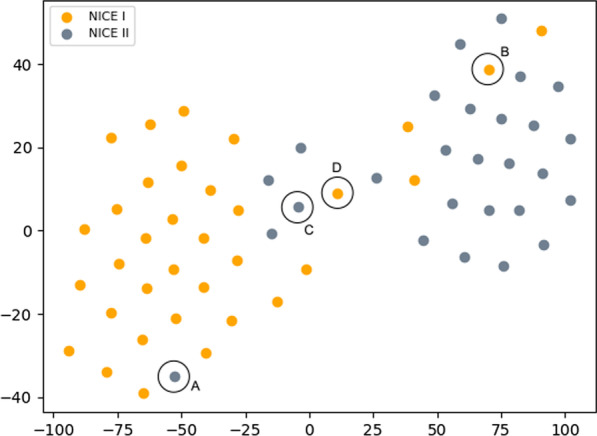
t-SNE embeddings [58] into 2D of the polyp images using the DTD trained encoder. The highlighted data points will be subject of a discussion

**Fig. 7:**
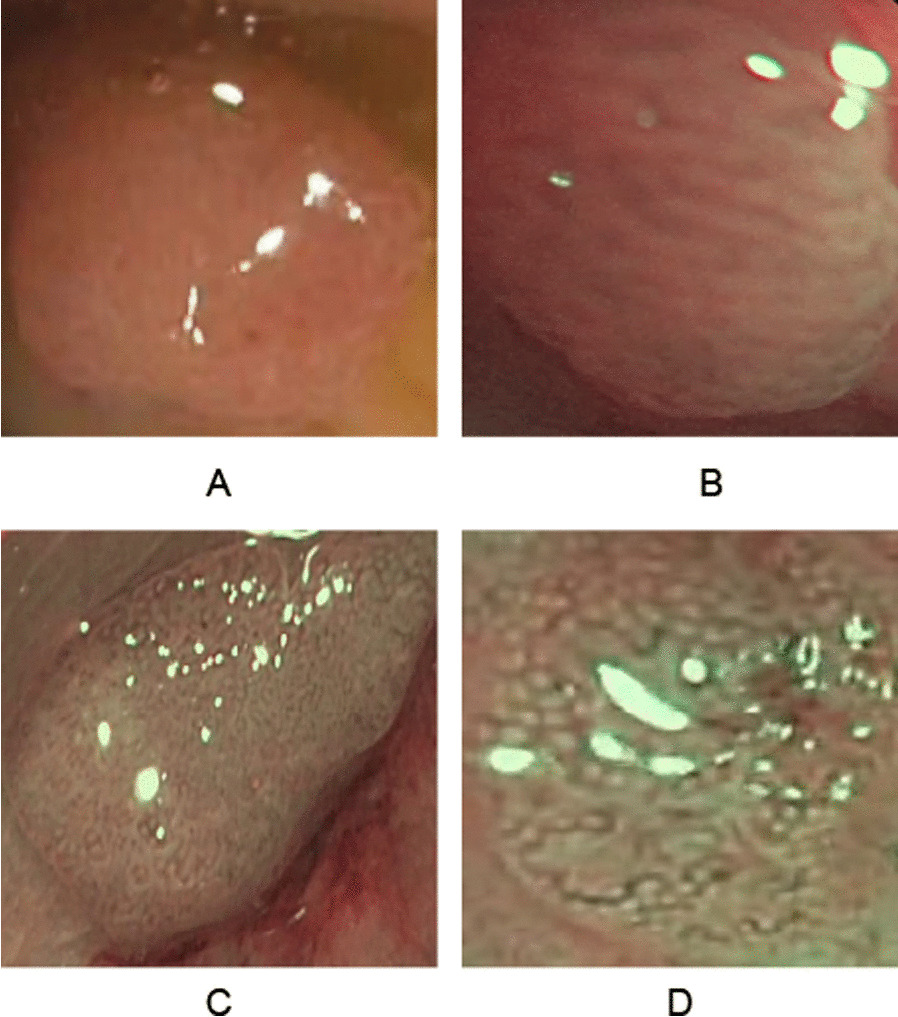
Samples of misclassified polyps of our system. The images **A** and **C** belong to the class NICE II. The images **B** and **D** appertain to class NICE I

This section will conclude the NICE classification with a short error analysis of the developed classification system.

The overall quality of the learned embedding can be seen in Fig. 6, which displays the t-SNE projections [58] into 2D of the embeddings generated by the DTD pre-trained encoder model. The projections reveal that the different NICE classes form two distinct clusters in the latent space, which possess however an overlapping zone, which reflects the classification performances given in Table 6.

We will now consider two kinds of problematic embeddings to gain further insights into the performance. Firstly, we consider two data points embedded well into the clusters of the wrong NICE class. The data points are denoted with A and B in Fig. 6 and in Fig. 7, where they are depicted in the upper row. As shown in Fig. 7, the image A is heavily blurred, such that its surface appears feature less. Note, that image A has also not been taken with the NBI-light activated. With the surface patterns not discernible, the homogeneous polyp has been embedded into the NICE I cluster of the latent space. Similarly, polyp B’s surface exhibits discernible tubular structures, which have likely been picked up by the encoder and led to an embedding into the NICE II cluster of the latent space.

Secondly, we will consider two polyps that populate the overlapping zone of the two latent clusters. The polyps concerned are denoted C and D in both Figs. 6 and 7. Both polyps display a pronounced surface texture and rich patterns. While in both cases, the features of their correct NICE class dominate the patterns (tubular in case of polyp C and spotted in D), both polyps display at close inspection also structures of the respective other NICE class.

We conclude from the presented error analysis that the NICE classification system facilitated by the polyp encoding neural network presented in this paper succeeds at generating semantically viable representations of polyps and embedding the polyps into a well-structured latent space apt for downstream usage in classification.

### Paris classification

For the Paris classification we compare two additional state-of-the-art algorithms to our approach for a fair comparison. For the comparison, we are using BiT-R152x4, and EfficientNet-B7. BiT-R152x4 and EfficientNet-B7 are both CNN architectures. Our model (ViT-L-16) with different learning rates, data augmentation methods, and dropout rates. This will help decide which hyperparameters and settings are needed for each model to train the best possible polyp classifiers.

#### Experimental design

For the evaluation of the Paris classification the images were divided into training, validation, and testing data sets based on the number of different polyps, with approximately 70 % of the polyp images from the SUN Colonoscopy Video data set being used for training, 15 % for validation, and 15 % for testing. The sun data set was thereby split in cases so that there is no polyp training data in which the same case would also be in the test data. The final test data consist of the 15% of polyps in the SUN data set split in cases and 15% of our own data set also split in individual cases.

Transfer learning models were used for training, pre-trained on existing data sets and refined for the polyp classification task. BiT-R152x4 and ViT-L-16 are used with the weights pre-trained on ImageNet-21k. ViT-L-16 was also finetuned on the ILSVRC-2012 data set [35, 37, 38]. In addition, EfficientNet has the special case that training can proceed in two phases. First, all weights in the network are frozen and only the last layers are adjusted. The second phase is optional and offers training in the deeper layers. For this work, both methods were used and the best results were presented.

Finding the correct hyperparameters for the models is essential for the accuracy of the models. Therefore, different parameters and settings were trained and tested for each model. The related results are presented in the ablation study subsection. For this purpose, this paper tested and selected different learning and dropout rates. Furthermore, different data augmentation methods were additionally tested to boost the performance of the models.

In addition to the different dropout rates and data augmentation, the early stopping method was used to avoid overfitting and long training times. For Big Transfer, training was stopped after seven epochs without improvement, while for EfficientNet, training was stopped after 20 epochs without improvement. For our model, the training was stopped after 11 epochs.

#### Evaluation

The evaluation is done via the F1-score and the accuracy. The F1-score describes the harmonic mean of precision and recall. The F1-score, the accuracy, the recall and precision are shown in following equations:

$$\begin{aligned} \text {Precision}= & {} \frac{TP}{TP+FP} \;\;\;\; \text {Recall} = \frac{TP}{TP+FN}\\ F_1= & {} \frac{2*\text {Precision}*\text {Recall}}{\text {Precision}+\text {Recall}} = \frac{2*TP}{2*TP+FP+FN}\\ Accuracy= & {} \frac{TP+TN}{TP+TN+FP+FN} \end{aligned}$$

We count an annotation as true positive (TP) if the classification of our prediction and GT do have the same label. If a polyp is predicted in a wrong class but the polyp is another class we count it as a false positive (FP). We calculate the TP, FP, true negatives (TN), false negatives (FN) for every class and calculate the scores according to the equations above.

For the testing BiT-R152x4 from Big Transfer, our model using ViT-L-16 from Vision Transformer, and B7 from EfficientNet were tested. The results are illustrated in the table below:

**Table 9:** Test results of each model on two different test data sets, the SUN Colonoscopy Video data set and our own data set (EndoData) [11]

Model	Data set	Acc	Pre	Rec	F1
BiT-R152x4	SUN	80.45	69.57	77.25	73.21
	EndoData	76.31	76.24	72.28	74.20
EfficientNet-B7	SUN	84.25	72.82	**80.27**	76.36
	EndoData	73.94	72.11	71.01	71.46
Ours	SUN	**89.35**	**84.76**	79.10	**81.28**
	EndoData	**87.42**	**80.09**	**78.83**	**79.45**

All values are given in %. Bold values are indicating the highest value of a column on the given data set

Table 9 shows that our approach using a transformer architecture outperforms the two other CNN approaches in nearly all metrics. Especially on the harder-to-classify EndoData [11]. The improvement from BiT-R152x4 to our model shows an accuracy of 76.31% to 87.42 %. A significant approvement considering our approach compared to the CNN approach. Nevertheless, the EfficientNet-B7 algorithm achieves a minimal improvement considering the recall on the SUN data set with an increase from 79.10 % to 80.27 % compared to our approach. As shown in Table 2, comparing these algorithms to the published literature in the domain is challenging because the algorithms are evaluated on different data sets and using different classes. Nevertheless, Bour et al., which is the best approach using three classes, achieved an accuracy of 87.1 % [4] on their test data set. With our model, we are surpassing this accuracy by 2.04 %. Nevertheless, in the paper of Bour et al. [4], 785 different polyps are used for training and validation, and the authors did not specify the amount and composition of the test data. Therefore, it is hard to make a fair comparison between the algorithms.

To further elaborate on the results of our model we computed the accuracy, precision, recall and F1-score for every Paris class individually. The results are shown in Table 10. For the accuracy the results indicate that classes Is and Ip are best classified by the model.

**Table 10:** In this figure, the test results of our model on the SUN Colonoscopy Video data set are shown for each Paris class individually. All values are given in %

Paris class	Acc	Pre	Rec	F1
Is	92.97	91.87	93.27	92.56
Ip	94.30	90.66	55.64	68.96
Isp	85.94	68.84	42.41	52.49
IIa	84.43	78.90	76.27	77.56
Mean	89.35	84.76	79.10	81.28

#### Ablation study

In this section, we present the results of the BiT-R152x4, EfficientNet-B7, and our model with different learning rates, data augmentation methods, and dropout rates. This will help decide which hyperparameters and settings are needed for each model to train the best possible polyp classifiers.

*Learning rate* To find a suitable learning rate for each model, the models were trained and tested with different learning rates. All models have, if applicable, a dropout rate of 0.5. For the data augmentation, our model and BiT-R152x4 were set to random flipping, while the EfficientNet-B7 results were computed with the combination of random flip, random rotation and random contrast. Table 11 shows the results for each model considering different learning rates. In addition, the time of one training epoch per minute and the required number (#) of epochs until reaching the best accuracy on the validation data set are given.

**Table 11:** Results on the validation data set considering different learning rates

Model	Learning rate				Val-acc	Training speed	
	0.01	0.001	0.0001	0.00016		Min/Epoch	#Epochs
BiT-R152x4	$\checkmark$				0.7890	$\approx$ 30	4
		$\checkmark$			**0.8213**	$\approx$ 30	8
			$\checkmark$		0.8140	$\approx$ 30	10
				$\checkmark$	0.8156	$\approx$ 30	10
EfficientNet-B7	$\checkmark$				0.7903	$\approx$ 5.7	6
		$\checkmark$			**0.8212**	$\approx$ 5.7	10
			$\checkmark$		0.7924	$\approx$ 5.7	30
				$\checkmark$	0.7969	$\approx$ 5.7	28
Ours	$\checkmark$				0.4668	$\approx$ 3	19
		$\checkmark$			0.5938	$\approx$ 3	23
			$\checkmark$		0.8242	$\approx$ 3	10
				$\checkmark$	**0.8950**	$\approx$ 3	8

Bold values are indicating the highest value of a column for the given model

Thereby, the results provide the first indications that for the CNN models BiT-R152x4 and EfficientNet-B7, the best results are obtained with the learning rate of 10$^{-3}$. Our model achieved better results with a lower learning rate. In addition, this required less time for one training epoch since the computational effort is lower for the Vision Transformer compared to the CNN models [35]. Another interesting aspect of the results in Table 11 is that for the CNN methods, the number of epochs increases when decreasing the learning rate, but for our transformer model, considering the first two learning rates of 0.01 and 0.001, the number of epochs is decreasing. This is contradictory and could be attributed to the fact that it is hard to learn for the transformer model with these learning rates and therefore, the training goes longer than it should. For the subsequent analysis to investigate data augmentation and dropout, the learning rate that provided the best validation accuracy in Table 11 was used for each model.

*Data augmentation*

In the second step of this analysis, various data augmentation methods were explored to adjust the models to best fit the polyp classification. Data augmentation helps combat overfitting and can create critical diversity in a data set. The increased diversity in the training data set improved the performance. The data augmentation methods used for this training are random flipping (random flip) or rotating the images (random rotation), and changing the contrast (random contrast). Table 12 presents the obtained training results considering different augmentation techniques.

**Table 12:** Results on the validation data set considering different data augmentation methods

Model	Data augmentation			Acc
	random flip	random rotation	random contrast	
BiT-R152x4				0.8155
	$\checkmark$			**0.8213**
	$\checkmark$		$\checkmark$	0.4543
	$\checkmark$	$\checkmark$		0.7968
	$\checkmark$	$\checkmark$	$\checkmark$	0.4469
EfficientNet-B7				0.7551
	$\checkmark$			0.7903
	$\checkmark$		$\checkmark$	0.7936
	$\checkmark$	$\checkmark$		0.8091
	$\checkmark$	$\checkmark$	$\checkmark$	**0.8212**
Ours				0.7930
	$\checkmark$			**0.8950**
	$\checkmark$		$\checkmark$	0.8210
	$\checkmark$	$\checkmark$		0.8242
	$\checkmark$	$\checkmark$	$\checkmark$	0.6016

Bold values are indicating the highest value of a column for the given model

The table shows that all models benefit from data augmentation. Training runs without data augmentation gave much worse results. This indicates that data augmentation is important for polyp classification. Especially the random horizontal and vertical flipping of the images seems to have a great effect for polyp classification. For the subsequent analysis to investigate dropout, the data augmentation that provided the best validation accuracy in Table 12 was used for each model. Random flipping and changing the contrast had different effects on the models. EfficientNet provided improved performance to 82.12 %. The other options in combination with flipping caused deterioration of the results for our model and BiT-R152x4. Nevertheless, their results achieved increased validation accuracy by random flipping alone. 89.50 % for our model and 82.13 % for BiT-R152x4.

*Dropout* Dropout is a regularization technique to avoid overfitting on the data set. As a further step, this section experiments with different dropout rates to make the models less susceptible to overfitting and thus achieve better values on the validation data set. With one exception for BiT-R152x4, dropout rates of 0.4, 0.5, and 0.6 were tested on the remaining models. The authors of BiT-R152x4 did not use dropout to avoid overfitting, but attempted to train stable models using the learning rate schedule method [37]. In the learning rate schedule method, no fixed learning rate is set for training, but varying learning rates are used. For example, at the beginning of the training, a large learning rate is used to move the gradient faster towards the minimum. Then the learning rate is decreased during training so that at the end the gradient does not skip the minimum. This results in reaching the minimum faster and the model gains higher accuracy.

**Table 13:** Results on the validation data set considering different dropout rates

Model		Dropout rate		Val-acc
	0.4	0.5	0.6	
EfficientNet-B7	$\checkmark$			0.8094
		$\checkmark$		**0.8212**
			$\checkmark$	0.7908
Ours	$\checkmark$			0.8593
		$\checkmark$		**0.8950**
			$\checkmark$	0.8513

Bold values are indicating the highest value of a column for the given model

BiT-R152x4 did not use dropout and is therefore not included in this table

The results in the 13 table show that the models produce solid results at all dropout rates, but show the best results at a dropout of 0.5 on the validation data set.

*Few-shot learning* As a last ablation, we want to briefly revisit the overall selection of the classification model and compare the performances of the Vision Transformer with the model underlying the few-shot learning system presented in the NICE classification section of this paper.

**Fig. 8:**
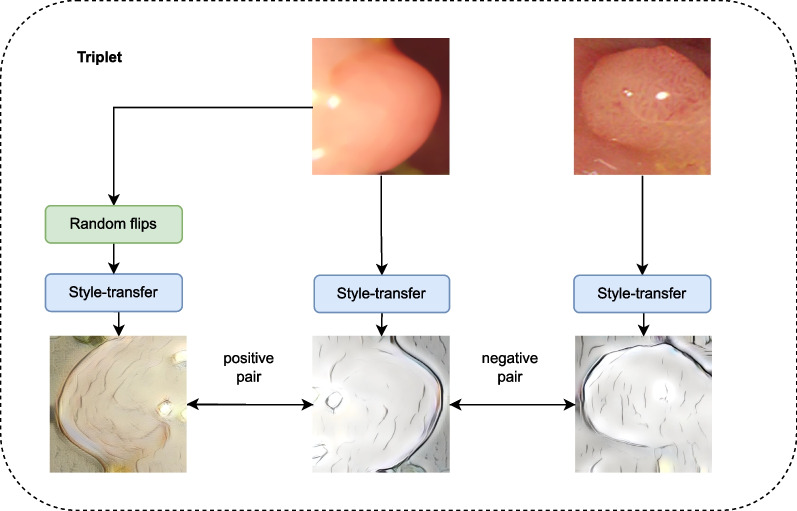
Triplet generation during the self-supervised pretraining for the Paris classification. The same style was used for the images of the negative pair, while different styles were used for the images of the positive pair

We deployed the outlined self-supervision approach, as the texture dataset DTD is inadequate for pretraining of a shape-centric classification task. As an augmentation engine facilitating the self-supervised pretraining, we deployed the style-transfer algorithm of [59], which provides a model capable of applying the style of arbitrary images to the content of another image. We selected the style-transfer as an augmentation step, as it allows the suppressing of most of the texture and style-related information of the original image and retains the structure and shape information as the main source of discriminative features. For the training, we selected pencil drawing styles, which we found to introduce almost no artificial texture to the images and highlight the structure and shape of the polyps in a very pronounced way. An overview of the deployed triplet generation is given in Fig. 8. The pretraining was again followed by a fine-tuning phase during which the triplets were constructed according to Paris class affiliation.

The configurations and parameters of the model and training remained identical to the setting described in the NICE classification sections of this paper.

Especially, the ResNet-18 has been retained as a feature extraction backbone and the SVM was used for the subsequent classification of the embeddings generated by the encoder.

The SUN data and the identical split of the 100 cases used in the preceding experiments involving the transformer were used to train and evaluate the model. Similarly to the pretraining of the self-supervised NICE classification system, we used a fully automated key frame selection pipeline to condense the training data down to 1081 images.

The system results are given in Table 14. As can be seen in the table, the system achieves high precision in the Paris class *IIa* and the minority classes *Ip* and *Isp*. However, the downside of the high precision is a weak recall, especially in the classes *Ip* and *Isp*, where all misclassified images were confused with the class *Is* or with *Is* and *Ip* in case of class *Isp*. The high precisions in the pedunculated classes allow the model to determine the presence of a pedunculation (*Ip* or *Isp*) with a $96.56\%$ precision. The low recall however is also reflected in the precision of the class *Is* under which many images showing protrusions are subsumed.

The proposed transformer displayed therefore the overall best results in the discussed task, albeit the metric-based system displays performances comparable to those of the other considered models, such as the EfficientNet, despite of the again considered scenario of little available data. Nevertheless, the approach using a state-of-the-art vision model above shows superior results considering the Paris classification.

**Table 14:** Results of the few-shot model in the SUN Colonoscopy Video data for each Paris class individually

Paris class	Acc	Pre	Rec	F1
Is	74.85	66.39	95.42	78.26
Ip	95.43	88.66	39.81	54.92
Isp	90.76	92.08	19.47	32.05
IIa	87.93	92.44	70.68	80.04
Mean	82.97	79.75	75.69	73.19

All values are given in %

## Discussion

In this chapter, we discuss the limitations and the explainability of the system. We primarily focus on wrong detections of the polyp classification system and discuss those system failures on the data sets. Additionally, we create heat maps showing the networks neural activation to gain deeper insight into the reasons for the classification results of the network. In this paper, two pre-trained CNN models as well as a pre-trained special transformer were used for the Paris classification. Especially the use of different data augmentation methods strongly improved the results of the models. Specifically, random image flipping seems to play an essential role in polyp characterization and should be looked at more closely in future research. This could be due to the reason that the Vision Transformer can understand and learn information about the whole image in the first layers of the model through the Attention layers. This presumably allows the model to better recognize the polyp features. CNNs, in turn, try to classify based on the locally recognized features [35], which profit from different augmentations.

### Limitations

First, assessing the test results, the distribution of images on the test data sets was unbalanced. Looking at the two test data sets, it is noticeable that the images with polyp types Is and IIa are particularly strongly represented, while the other classes are less represented. This may weaken the significance of the test results. However, the proportion of classes Ip and Isp in the training and validation data set is also low, and this may cause the models to classify these two classes moderately. This is due to the lack of labeled data sets for the polyp domain, which leads to the following limitation.

**Fig. 9:**
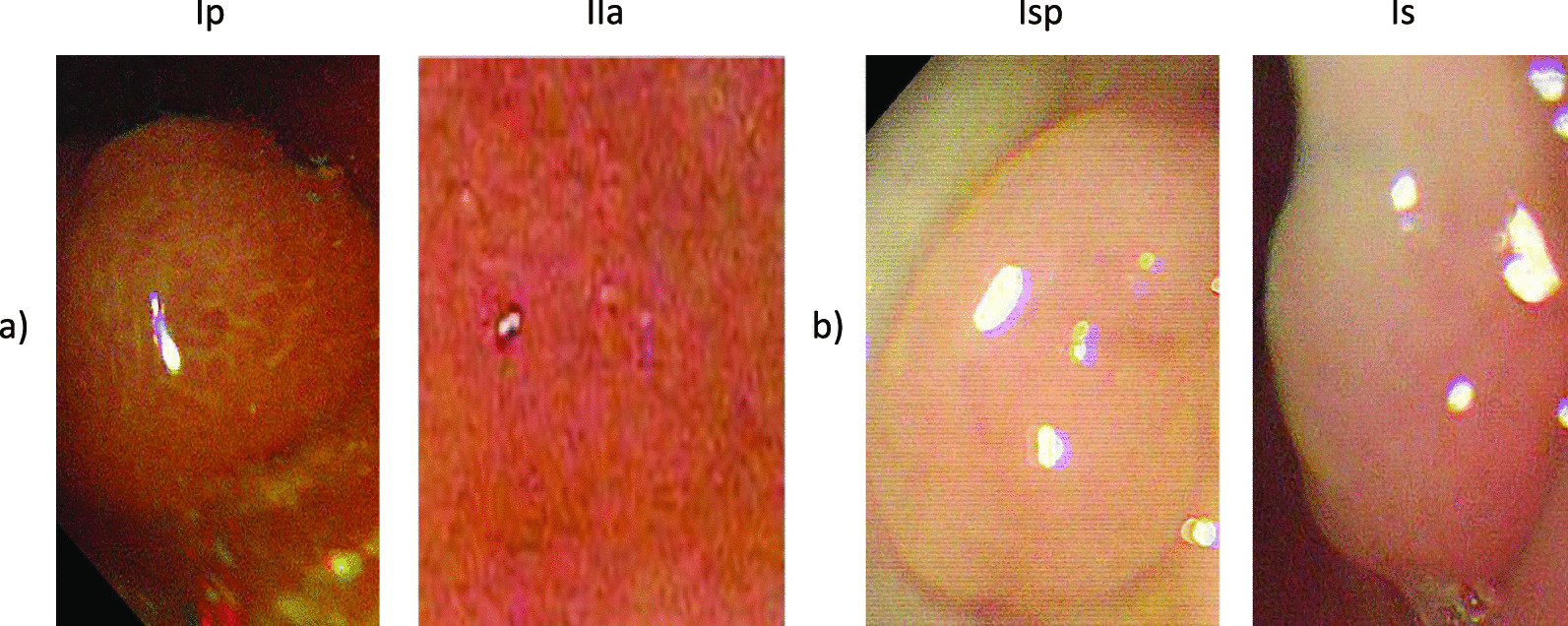
Model detection problems due to (**a**) difficult to detect polyps due to poor resolution and due to (**b**) the high similarity of the mixed form Isp class to Is. Images are taken from the SUN data set [32]

The lack of data is a significant problem, specifically in computational medical research, as a large amount of training data is required to build and train stable and accurate deep learning models. However, the number of annotated data sets, specially labeled polyp data sets for Paris classification, are severely limited. In addition, the existing polyp data sets still contain few polyp images for a deep learning task. For, e.g., the SUN Colonoscopy video data set [32], the data set consists of just 100 different polyps, of which nearly 70 are different polyps for training. This number tends to be too small to train a stable classifier. Therefore the diversity of polyps is missing. Moreover, the individual polyp cases of the data set consist of image frames of colonoscopy videos. This leads to the next problem, which may further impact the trained object recognition models. First, a colonoscopy video is many image sequences of one polyp. If we exclude the possible blur and distortion in the frames, the sequences consist of barely or slightly distinguishable images of polyps that are used to train the network. On the other hand, the videos are occasionally based on distant images of polyps, which were cropped and used again in this work based on the annotations. Thus, the data set used contains mostly small images, making them difficult to recognize, as shown by image section (a) in Fig. 9.

An additional obstacle in training the classifiers relates to the Paris classification. Since the SUN Colonoscopy Video data set contains polyp images for classes Ip, Isp, Is, and IIa, the object recognition models were examined to classify these four types. Here, it was noticeable that class Isp, the mixed form of Is and Ip, is difficult to identify for the classification models. Here, tests have shown that the mixed form is usually classified as one of the two primary forms due to the high similarity, as shown in an image section (b) in Fig. 9. Another reason for the confusion is the angle at which the image is acquired. Because a polyp is imaged from multiple sides during a colonoscopy, images of polyps are produced that cannot lead to a definite conclusion about the shape. For example, an image above of a pedunculated polyp (Ip) does not provide any information about the shape because, most likely, no pedicle can be seen. This problem mainly affects the classes Ip and Isp.

Lastly, extending the classification to all Paris classes would be very important. Since classes are missing and there is no “other” class, inherent errors are made when a polyp has a non-modeled class. To create a system with all classes, it would be necessary to construct bigger data sets in which those uncommon classes are highly represented.

### Heat maps for the Paris classification

**Fig. 10:**
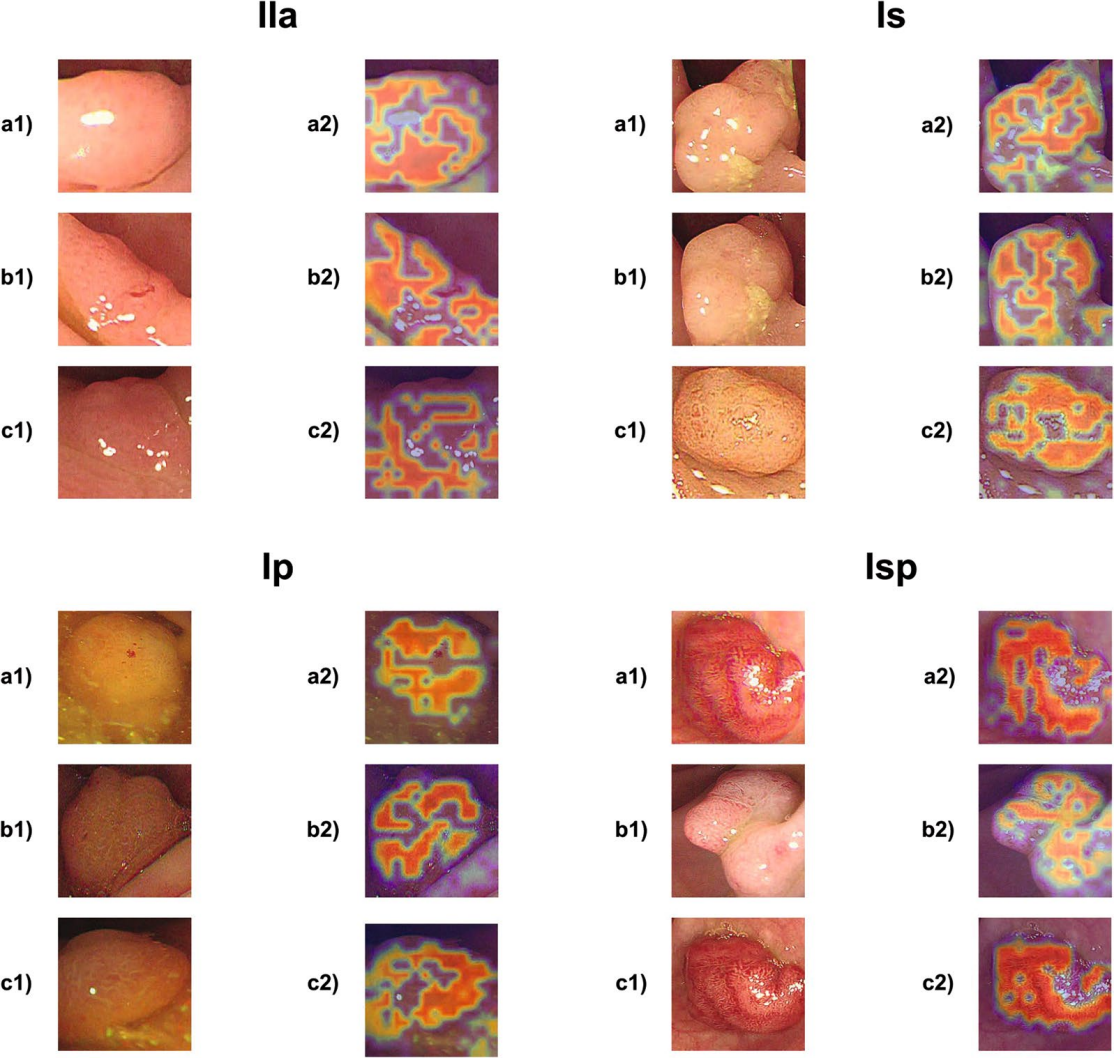
Heat maps for polyp classification. This figure illustrates the classifications of the model using the GRAD-CAM algorithm [60]. Thereby, pixels most relevant for the classification are marked in warm colors like red, and pixels less relevant for the neural network in cold colors like blue. Images are taken from the SUN data set [32]

In this section, we demonstrate the use of GradCAM to see what areas are essential for the network to classify a polyp. For this, we used GradCAM with Eigen smooth, a method to remove much noise in the heatmap. We picked three examples for each class to demonstrate the results (see Fig. 10). This paragraph presents a methodology to generate visual explanations for deriving insight into our polyp classification systems decisions using the Grad-CAM algorithm [60]. We follow the Checklist for Artificial Intelligence in Medical Imaging (CLAIM) [61].

Analyzing Fig. 10, throughout the examples, the network mostly looks at the polyp’s surface and not the background. Furthermore, there are gaps in the heat maps at areas of light reflections, which shows that the network can filter unnecessary information. Especially, for example, Isp with images c1) and c2) shows the AI ignores the background and the light reflections and only considers the structure of the polyp for the classification. In the Ip class, in image a1), we can see a red mark on the polyp. Even that mark is excluded and is not considered by the network, see image a2).

## Conclusion

In this paper, we show two novel automated classifications system assisting gastroenterologists in classifying polyps based on the NICE and Paris classification. We introduce a two-step process for the Paris classification: first, detecting and cropping the polyp on the image, and subsequently classifying the polyp with a transformer network. For the NICE classification, we designed a few-shot learning algorithm based on the Deep Metric Learning approach. The algorithm creates an embedding space for polyps, which allows classification from a few examples to account for the data scarcity of NICE annotated images in our database. Overall, our Paris classification system shows state-of-the-art results on a publicly available data set with an accuracy of 89.35 %, surpassing all papers in the literature. For the NICE classification, we achieve a competitive accuracy of 81.34 % demonstrating thereby the viability of the FSL approach in data-scarce environments in the endoscopic domain.

## Data Availability

The first data set used for the analysis of this article is available at the following link (http://sundatabase.org/). The second data set (EndoData) used during the analysis is available from the corresponding author on reasonable request.

## Abbreviations

CRC: Colorectal cancer
CNN: Convolutional neural network
CAD: Computer-aided detection
CADx: Computer-aided diagnosis
JSON: JavaScript Object Notation
AI: Artificial intelligence
SUN: Showa University and Nagoya University
WCE: Wireless Capsule Endoscopy
CEM: Context enhancement module
GAN: Generative Adversarial Network
FastCat: Fast Colonoscopy Annotation Tool
FPS: Frames per second
GPU: Graphical processing unit
R-CNN: Region based convolutional neural network
FSL: Few-shot learning
DTD: Describable Texture Data set
CLAIM: Checklist for Artificial Intelligence in Medical Imaging
Acc: Accuracy
Pre: Precision
Rec: Recall
Val: Validation
NBI: Narrow Band Imaging
NICE: NBI International Colorectal Endoscopic

## Footnotes

1 https://www.endoscopy-campus.com/en/classifications/polyp-classification-nice/.

2 https://www.endoscopy-campus.com/en/classifications/polyp-classification-nice/.

3 https://www.robots.ox.ac.uk/~vgg/data/dtd/.
